# Effects of Confounding Factors on Liver Stiffness in Two-Dimensional Shear Wave Elastography in Beagle Dogs

**DOI:** 10.3389/fvets.2022.827599

**Published:** 2022-01-27

**Authors:** Jinwoo Cha, Jayon Kim, Jaeeun Ko, Jaehwan Kim, Kidong Eom

**Affiliations:** Department of Veterinary Medical Imaging, College of Veterinary Medicine, Konkuk University, Seoul, South Korea

**Keywords:** two-dimensional shear wave elastography, liver stiffness, anesthesia, breathing, scanning approach, dog

## Abstract

**Background:**

Two-dimensional shear wave elastography (2D-SWE) is a powerful technique that can non-invasively measure liver stiffness to assess hepatic fibrosis.

**Purpose:**

This study aimed to identify the effects of confounding factors, including anesthesia, breathing, and scanning approach, on liver stiffness when performing 2D-SWE in dogs.

**Materials and Methods:**

Nine healthy Beagle dogs were included in this study. Hepatic 2D-SWE was performed, and liver stiffness was compared between conscious and anesthetized states, free-breathing and breath-holding conditions, and intercostal and subcostal approaches. For the anesthetized state, the breath-holding condition was subdivided into seven phases, which included forced-expiration (5 and 10 mL/kg), end-expiration (0 cm H_2_O), and forced-inspiration (5, 10, 15, and 20 cm H_2_O), and liver stiffness was compared among these phases. Changes in liver stiffness were compared between intercostal and subcostal approaches according to breathing phases.

**Results:**

No significant difference was observed in liver stiffness between the conscious and anesthetized states or between the free-breathing and breath-holding conditions. No significant difference was noted in liver stiffness among the breathing phases, except for forced-inspiration with high airway pressure (15 and 20 cm H_2_O in the intercostal approach and 10, 15, and 20 cm H_2_O in the subcostal approach), which was associated with significantly higher liver stiffness (*p* < 0.05). Liver stiffness was significantly higher in the subcostal approach than in the intercostal approach (*p* < 0.05). Changes in liver stiffness were significantly higher in the subcostal approach than in the intercostal approach in all forced-inspiratory phases (*p* < 0.05).

**Conclusion:**

In conclusion, when performing 2D-SWE in dogs, liver stiffness is unaffected by anesthesia and free-breathing. To avoid inadvertent increases in liver stiffness, the deep inspiratory phase and subcostal approach are not recommended. Thus, liver stiffness should be interpreted considering these confounding factors.

## Introduction

Hepatic fibrosis, which often occurs as a consequence of chronic hepatitis, is a relatively common finding that affects 12% of dogs. For a definitive diagnosis of hepatic fibrosis in dogs, liver biopsy is warranted for histopathologic examination (1). However, the invasive nature of liver biopsy causes several complications such as excessive hemorrhage, post-biopsy pain, and shock (2). Accordingly, safe and non-invasive methods such as ultrasound (US) and magnetic resonance elastography are gaining popularity as assessment tools for hepatic fibrosis (3).

US elastography enables the quantitative measurement of liver stiffness based on deformation induced by forces exerted on a tissue (4). Based on the type of measured physical quantity, US elastography can be classified as strain elastography, acoustic radiation force impulse imaging, transient elastography, point shear wave elastography, and two-dimensional shear wave elastography (2D-SWE). 2D-SWE is a novel method that measures liver stiffness by using acoustic radiation force and inducing shear waves in multiple focal zones. Both anatomical and stiffness information of the liver can be acquired using shear-wave propagation and color maps superimposed on B-mode images of the liver parenchyma (4–6). In humans, 2D-SWE is frequently performed in patients with diffuse liver disease for the diagnosis and staging of hepatic fibrosis (3–5, 7). Recent veterinary literature has also suggested that 2D-SWE can be used for predicting the presence of clinically relevant hepatic fibrosis in dogs (8).

When performing 2D-SWE, liver stiffness does not solely reflect hepatic fibrosis, as multiple confounding factors can artifactually increase liver stiffness. Accordingly, it is crucial to consider all potential confounding factors that may affect liver stiffness. Common confounding factors in human elastography include the probe, scanning approach, region of interest (ROI), liver lobe, positioning, fasting, anesthesia, and breathing (3, 7, 9). A recent veterinary study using 2D-SWE assessed the effects of ROI, liver lobe, and scanning approach on liver stiffness (10). However, key confounding factors such as anesthesia, breathing, and scanning approach have not been comprehensively evaluated in dogs to date.

Anesthesia or sedation is administered to young children or dogs with low compliance to reduce unintended movements, which cause invalid measurements of liver stiffness (10, 11). In humans, the effects of anesthesia on liver stiffness are taken into consideration when measuring liver stiffness (9); however, these effects tend to be overlooked in veterinary medicine. Moreover, two different aspects of breathing should be considered: breathing motion artifacts and breathing phase (3). Breathing motion artifacts are considered in uncooperative children who cannot hold their breath and therefore breathe freely. For this reason, various human and phantom studies comparing liver stiffness between free-breathing and breath-holding conditions have been conducted, but the results have been conflicting (12–17). Furthermore, breathing phase, including inspiration and expiration, can significantly impact liver stiffness. Nevertheless, results have been controversial, with some studies reporting higher liver stiffness in inspiration than in expiration (3, 18–20), and other studies reporting the opposite effect (21, 22).

Scanning approach, including intercostal and subcostal approaches, is another confounding factor that can affect liver stiffness (7). Various elastography studies have been performed in humans and dogs to compare the intra- and inter-observer agreements and liver stiffness between intercostal and subcostal approaches (5, 10, 23). However, the interaction between scanning approach and breathing and its effects on liver stiffness have yet to be reported.

Therefore, this study aimed to evaluate the effects of confounding factors on liver stiffness by investigating the impact of anesthesia, breathing, and scanning approach when performing 2D-SWE in dogs.

## Materials and Methods

### Animals

Nine intact male Beagle dogs with a mean age of 1.9 years (range, 1–8 years) and a mean weight of 10.0 kg (range, 8.7–10.9 kg) were used in this experimental study. All dogs were confirmed to be clinically healthy based on physical examination, a complete blood panel including hematocrit and blood chemistry, radiography, abdominal ultrasonography, and echocardiography. The decision to include or exclude the dogs was determined by a veterinarian with 2 years of experience in veterinary imaging. The study procedures were approved by the Institutional Animal Care and Use Committee of Konkuk University (approval no. KU 21127).

### Hepatic 2D-SWE

Prior to the examination, the dogs were fasted for at least 12 h. Conventional B-mode ultrasonography and 2D-SWE were performed using an ultrasound scanner (Aplio i800, Canon Medical Systems, Tochigi, Japan) and a 3.8-14.0 MHz linear-array transducer (PLT-1005BT, Canon Medical Systems). The right lobe of the liver was selected as the target region for examination. After clipping the target region and applying a sufficient amount of ultrasonographic gel, B-mode US images of the liver were acquired with minimal compression of the probe. In the B-mode images, the intrusion depth was 5 cm and a rectangular 1 ×1 cm field of view (FOV) was set at a minimum of 10 mm below the liver capsule to avoid possible reverberation artifacts. A multi-shot mode of 2D-SWE, which emits continuous push pulses from the probe and enables the selection of a single image, was performed. Within the selected image, a round ROI with a 6-mm diameter was placed in the FOV while avoiding the diaphragm, large vasculature, and ductal structures. These measurements were repeated with different FOV and ROIs until seven valid measurements were obtained. The mean of the obtained data was presented as Young's modulus (kPa), which was automatically calculated by the installed software *via* a conversion formula using shear wave velocity (m/s).

**Figure 1:**
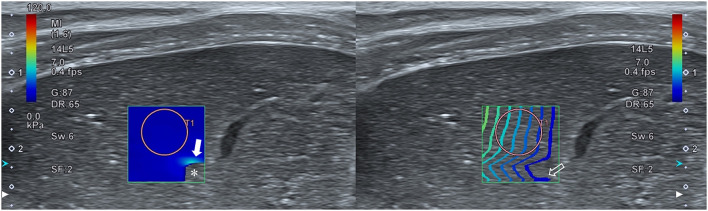
Reliability of hepatic two-dimensional shear wave elastography. A dual-screen mode displays a color map and propagation map on the left and right screens, respectively. Regions with a uniform blue color on the color map and parallel lines with a constant interval on the propagation map represent high reliability. Regions with no color (asterisk) or a focal heterogeneous color (closed arrow) on the color map and distorted, chaotic lines (open arrow) on the propagation map represent low reliability.

In accordance with recommended guidelines for elastography in humans and based on results from previous studies in dogs, the reliability of the data was confirmed using color and propagation maps, which were displayed in dual-screen mode. The color map represented tissue stiffness, with blue and red representing the lowest and highest stiffness, respectively. The regions of uniform blue color were considered to be highly reliable, while the non-color-coded regions and focal heterogeneous green color indicated low reliability and were therefore not measured. The propagation map depicted shear wave arrival times at different locations *via* contour lines. Parallel and straight lines with constant intervals were regarded as highly reliable data, while distorted and chaotic lines were considered to indicate unreliable data (8, 10) (Figure 1).

### Protocols for Investigating Confounding Factors

Hepatic 2D-SWE was performed in both the conscious and anesthetized states. For the anesthetized state, general anesthesia was induced with propofol (0.6 mg/kg, Provive™ injection 1%; Claris Injectables Ltd., Ahmedabad, India) and maintained with 2.0% isoflurane (Terrell, Piramal Critical Care, Inc., Bethlehem, PA, USA) and oxygen (1.0 L/min). Heart rate, blood pressure, oxygen saturation, and end-tidal CO_2_ were monitored continuously during anesthesia.

**Figure 2:**
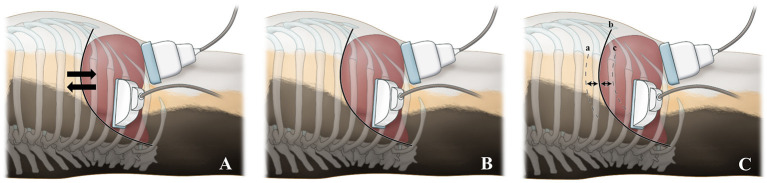
Illustration of diaphragmatic and hepatic movements in various breathing conditions and probe positions according to the scanning approaches. During both the conscious and anesthetized free-breathing conditions **(A)**, the diaphragm and liver move freely during spontaneous breathing. During the conscious breath-holding condition **(B)**, the diaphragm and liver do not move and are temporarily held in a static position, as breathing is halted by holding the nose and mouth or by blowing air into the nose. During the anesthetized breath-holding condition **(C)**, the diaphragm and liver do not move and are held in a static position. The diaphragmatic positions are illustrated in forced-expiration (cranial; 5 and 10 mL/kg) (a), end-expiration (neutral; 0 cm H_2_O) (b), and forced-inspiration (caudal; 5, 10, 15, and 20 cm H_2_O) (c). For the intercostal approach, the probe was placed parallel to the ribs in the 10–12th intercostal space. For the subcostal approach, the probe was positioned beneath the costal arch.

**Figure 3:**
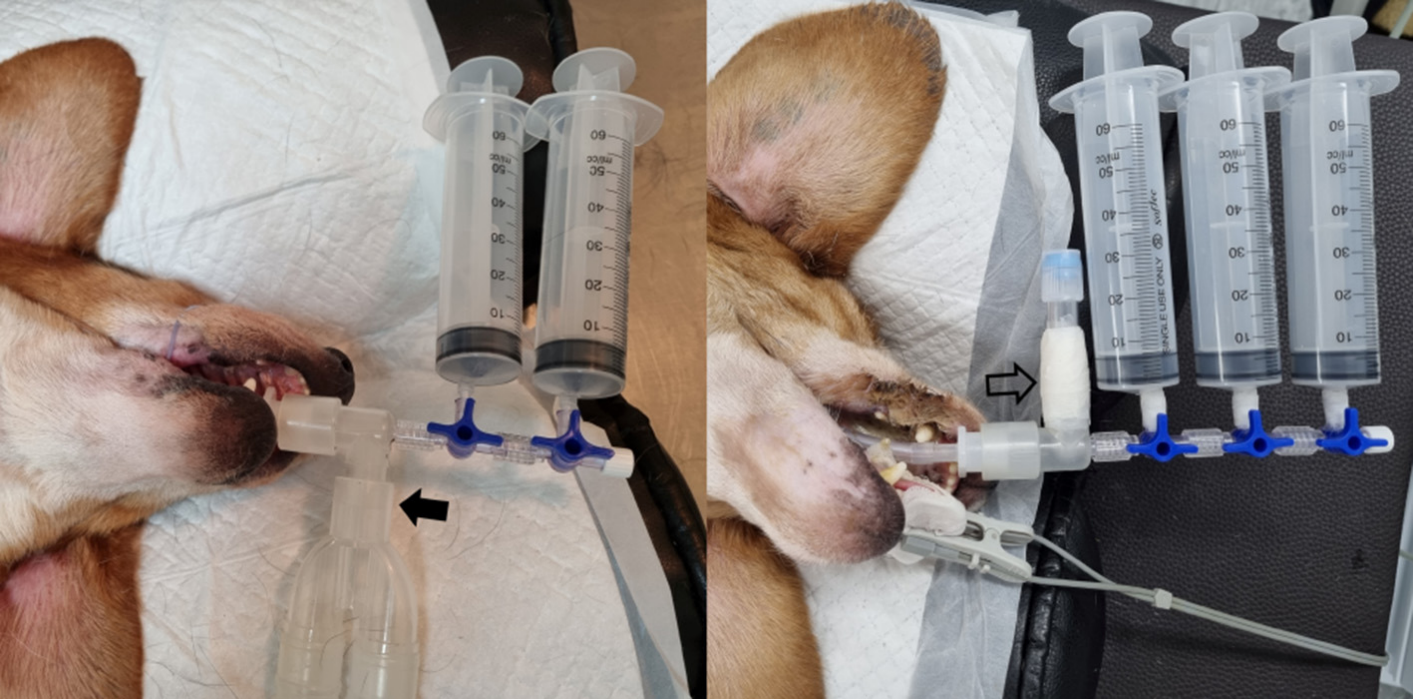
Instrument settings for inducing forced-expiration under anesthesia during hepatic two-dimensional shear wave elastography. After connecting 50-cc syringes to the endotracheal tube with a three-way valve, the section of the tube connected to the anesthesia machine (arrow) is disconnected and blocked (open arrow) just before drawing out the air *via* syringes.

Liver stiffness was measured in two breathing conditions, which included free-breathing and breath-holding. In both the conscious and anesthetized states, 2D-SWE during free-breathing was examined when the dogs were in a stable state with normal breathing. In the conscious state, 2D-SWE during breath-holding was performed after stopping the air flow by holding the nose and mouth of the dogs or by blowing air into the nose abruptly. In the anesthetized state, the breath-holding condition was subdivided into seven breathing phases, including forced-expiration (5 and 10 mL/kg), end-expiration (0 cm H_2_O), and forced-inspiration (5, 10, 15, and 20 cm H_2_O) (Figure 2). For forced-expiration, 50-cc syringes connected to the endotracheal tube were pulled manually, drawing the air out from the airway and inducing expiration. The number of syringes used was selected based on the functional residual capacity of the lung in accordance with the body weight of each dog (5 and 10 mL/kg). The baseline used in this study was lower than that reported in the veterinary literature to ensure the safety of the dogs (24). To prevent additional air from entering the lungs during 2D-SWE, the section of the tube connected to the anesthesia machine was blocked just prior to the induction of forced-expiration (Figure 3). Other breathing phases were manually controlled by manipulating the reservoir bag of the anesthesia machine. End-expiration was obtained by hyperventilating the dogs to induce short-term apnea resulting from decreased end-tidal CO_2_ pressure and thus achieving an airway pressure of 0 cm H_2_O. Forced-inspiration was induced by compressing the reservoir bag manually and achieving an airway pressure of 5, 10, 15, and 20 cm H_2_O.

Two different US scanning approaches were used: intercostal and subcostal approaches (Figure 2). For the intercostal approach, the dogs were positioned in left lateral recumbency and the probe was placed parallel to the ribs in the 10–12th intercostal space. For the subcostal approach, the dogs were positioned in dorsal recumbency and the probe was placed beneath the costal arch.

### Statistical Analysis

Statistical analysis was conducted with commercially available software (SPSS®, version 25, IBM Corp., Armonk, NY, USA; and Excel, Microsoft Corp., Redmond, Washington, USA) by one veterinarian. All measured data were presented as means and standard deviation (SD). The coefficient of variation (CV) was calculated for all variables by dividing each SD by the mean. A CV of <30% was considered clinically reliable. The normality of the obtained data was confirmed using the Shapiro–Wilk test. For normally distributed data, a paired *t*-test was performed to assess the significance of differences in liver stiffness between conscious and anesthetized states, free-breathing and breath-holding conditions, and intercostal and subcostal approaches. One-way repeated-measures analysis of variance followed by Bonferroni correction for multiple comparisons were performed to compare liver stiffness among breathing phases. For both scanning approaches, changes in liver stiffness were calculated by subtracting liver stiffness in end-expiration 0 cm H_2_O from the stiffness in each breathing phase. A *p* < 0.05 was considered statistically significant.

## Results

### Conscious and Anesthetized States

In the free-breathing condition, no significant difference was observed in liver stiffness between the conscious and anesthetized states for both scanning approaches (*p* = 0.169 for intercostal approach and *p* = 0.533 for subcostal approach) (Table 1).

**Table 1:** Liver stiffness in intercostal and subcostal approaches.

	**Intercostal**	**Subcostal**	***p*** **-value**
FB	7.25 ± 0.33 kPa	7.80 ± 0.43 kPa	0.003
BH	7.19 ± 0.36 kPa	7.76 ± 0.44 kPa	0.004
AN_FB	7.33 ± 0.38 kPa	7.90 ± 0.35 kPa	0.001
AN_FE 10 mL/kg	7.26 ± 0.59 kPa	7.75 ± 0.38 kPa	0.040
AN_FE 5 mL/kg	7.27 ± 0.61 kPa	7.83 ± 0.43 kPa	0.010
AN_EE 0 cm H_2_O	7.44 ± 0.54 kPa	7.84 ± 0.45 kPa	0.007
AN_FI 5 cm H_2_O	7.47 ± 0.63 kPa	8.14 ± 0.54 kPa	0.002
AN_FI 10 cm H_2_O	7.85 ± 0.65 kPa	8.99 ± 0.73 kPa	0.001
AN_FI 15 cm H_2_O	8.48 ± 0.59 kPa	10.02 ± 1.03 kPa	0.006
AN_FI 20 cm H_2_O	9.34 ± 0.77 kPa	10.87 ± 1.01 kPa	0.004

*All data are presented as mean ± standard deviation*.

*p < 0.05 is considered statistically significant*.

*AN, anesthetic state; FB, free-breathing; BH, breath-holding; FE, forced-expiration; EE, end-expiration; FI, forced-inspiration*.

### Free-Breathing and Breath-Holding Conditions

No significant difference was observed in liver stiffness in the conscious state between free-breathing and breath-holding conditions for both scanning approaches (*p* = 0.119 for intercostal approach and *p* = 0.190 for subcostal approach) (Table 1).

### Breathing Phases

**Figure 4:**
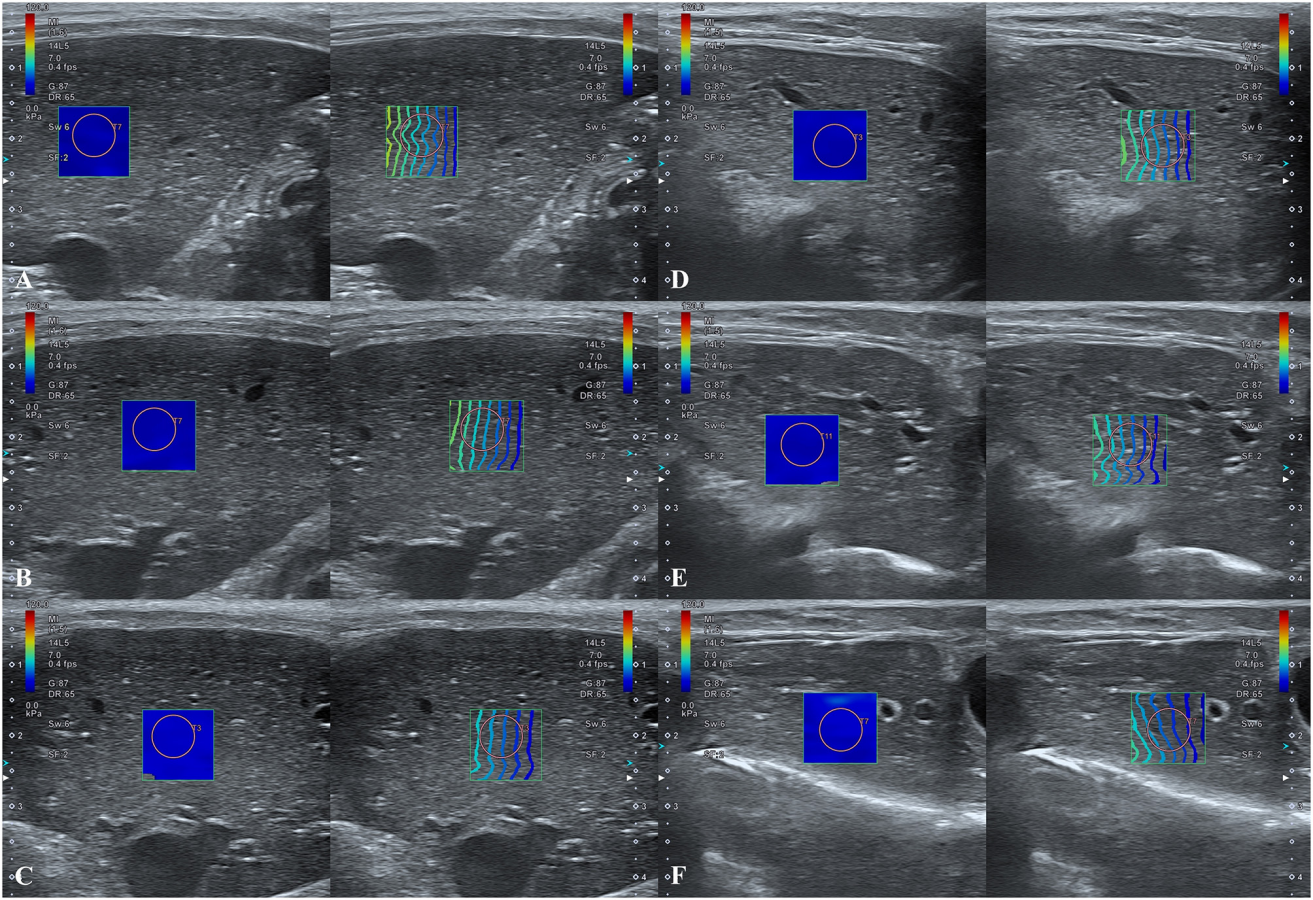
Hepatic two-dimensional shear wave elastographic images according to breathing phases. **(A–C)** Intercostal approach at **(A)** forced-expiration 10 mL/kg, **(B)** end-expiration 0 cm H_2_O, and **(C)** forced-inspiration 20 cm H_2_O. **(D–F)** Subcostal approach at **(D)** forced-expiration 10 mL/kg, **(E)** end-expiration 0 cm H_2_O, and **(F)** forced-inspiration 20 cm H_2_O. Note that the diaphragmatic movements according to breathing phases in 2D-SWE images are greater for the subcostal approach than for the intercostal approach.

**Figure 5:**
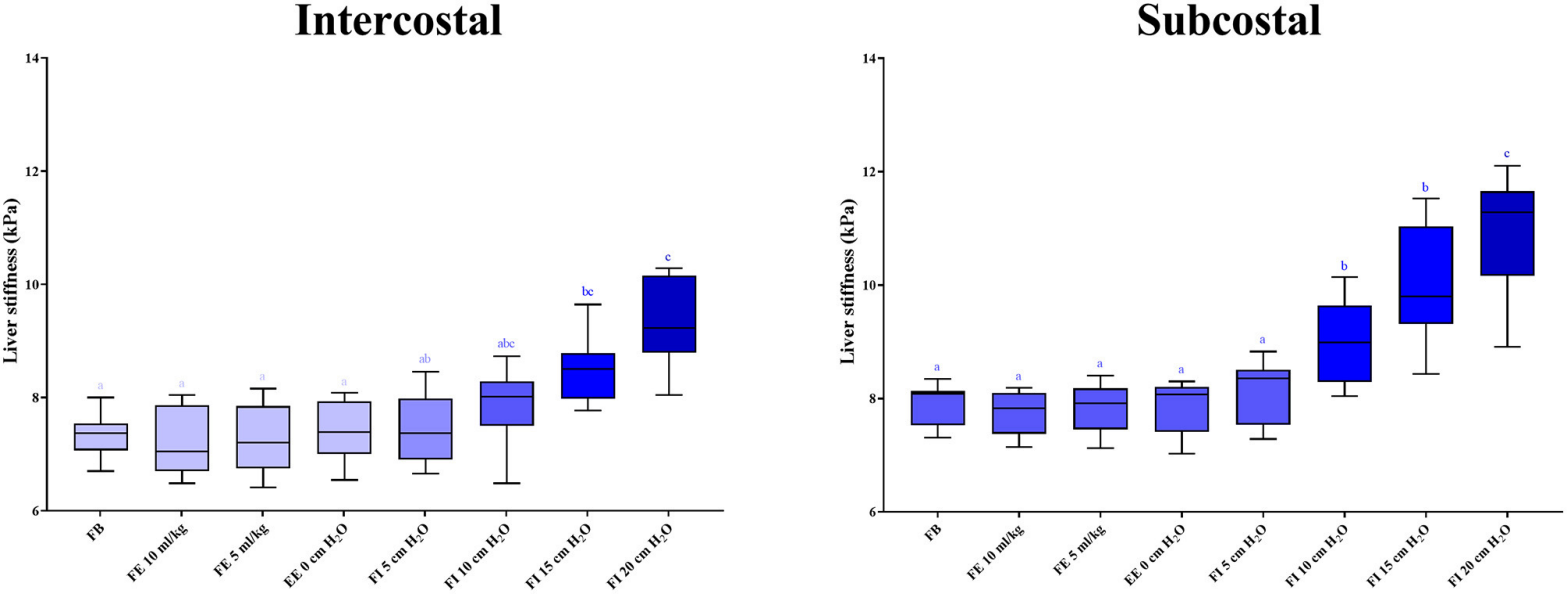
Box plots representing liver stiffness according to breathing phases. Boxes not sharing the same letters exhibit significant differences in liver stiffness (*p* < 0.05). Liver stiffness is significantly higher in forced-inspiration 15 and 20 cm H_2_O than in the other breathing phases in the intercostal approach. Liver stiffness is significantly higher in forced-inspiration 10, 15, and 20 cm H_2_O than in the other breathing phases in the subcostal approach. FB, free-breathing; FE, forced-expiration; EE, end-expiration; FI, forced-inspiration.

In the anesthetized state, 2D-SWE images exhibited a uniform blue color in the color maps and relatively thin and narrow contour lines in the propagation map during the expiratory phases. Images with a light-blue color in the color map and relatively thick and distant contour lines in the propagation map were acquired in several inspiratory phases, indicative of higher liver stiffness (Figure 4). In the intercostal approach, no significant differences were observed in liver stiffness among free-breathing, forced-expiration (5 and 10 mL/kg), end-expiration (0 cm H_2_O), and forced-inspiration (5 and 10 cm H_2_O). Liver stiffness was significantly higher in forced-inspiration 15 cm H_2_O than in free-breathing, forced-expiration (5 and 10 mL/kg), and end-expiration 0 cm H_2_O. Further, liver stiffness was significantly higher in forced-inspiration 20 cm H_2_O than in free-breathing, forced-expiration (5 and 10 mL/kg), end-expiration 0 cm H_2_O, and forced-inspiration 5 cm H_2_O. In the subcostal approach, there was no significant difference in liver stiffness among free-breathing, forced-expiration (5 and 10 mL/kg), end-expiration 0 cm H_2_O and forced-inspiration 5 cm H_2_O. Liver stiffness was significantly higher in forced-inspiration 10 and 15 cm H_2_O than in the other breathing phases, except for forced-inspiration 20 cm H_2_O. Liver stiffness was significantly higher in forced-inspiration 20 cm H_2_O than in all other breathing phases (Table 1, Figure 5).

### Intercostal and Subcostal Approaches

Liver stiffness across all breathing conditions and phases was significantly higher using the subcostal approach compared to the intercostal approach (Table 1). The CVs for all variables were <15% (range, 0.59–11.53%).

### Changes in Liver Stiffness According to Breathing Phases

**Figure 6:**
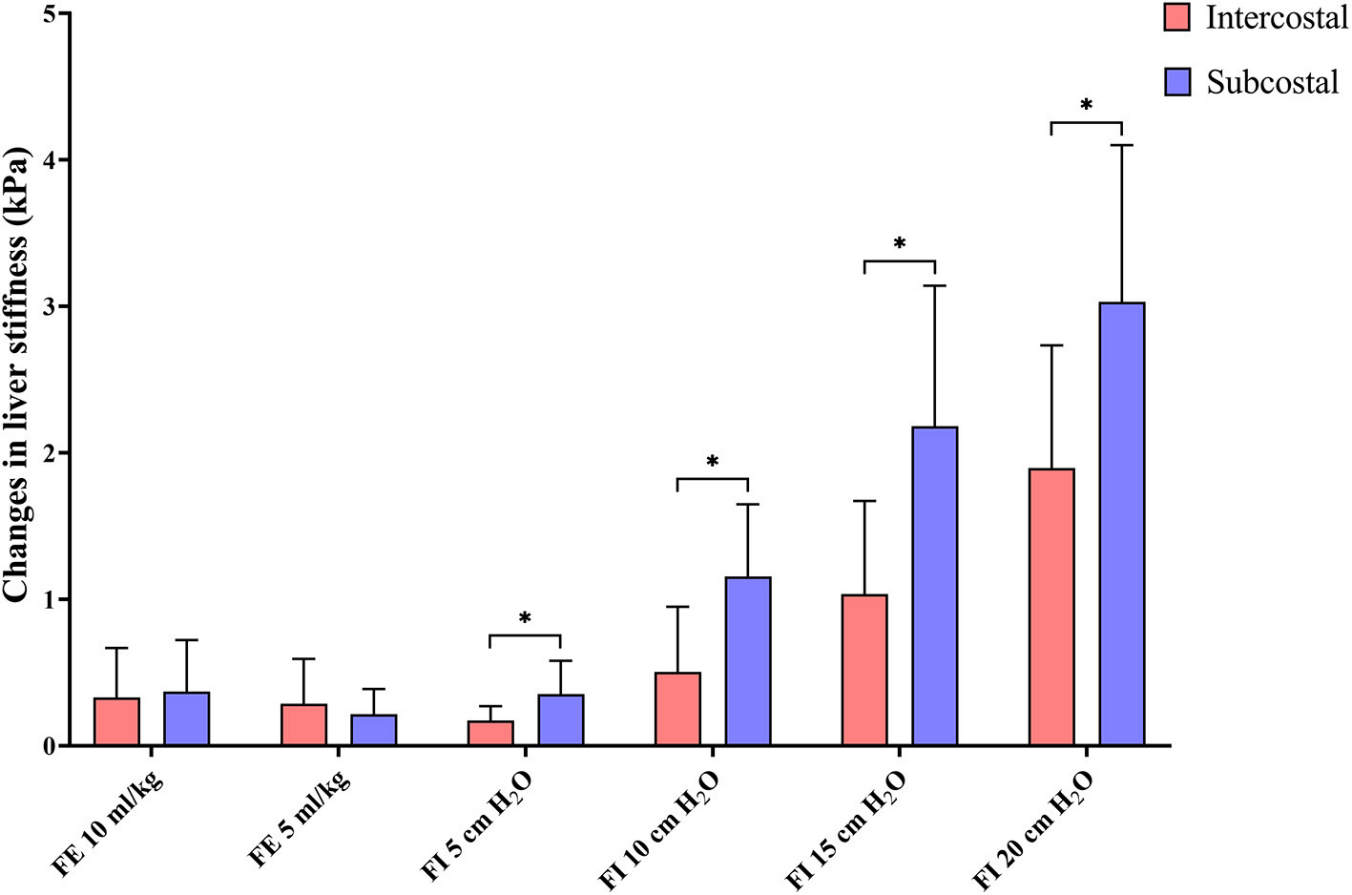
Bar graph representing the changes in liver stiffness. The changes in liver stiffness are calculated by subtracting liver stiffness in end-expiration 0 cm H_2_O from liver stiffness in each breathing phase. The bars are grouped by breathing phases, with red and blue representing the intercostal and subcostal approaches, respectively. Changes in liver stiffness are significantly higher in the subcostal approach than in the intercostal approach in all forced-inspiratory phases. *p < 0.05. FE, forced-expiration; FI, forced-inspiration.

The changes in liver stiffness were significantly higher in the subcostal approach compared to the intercostal approach in all forced-inspiratory phases (*p* < 0.05) (Table 2, Figure 6).

**Table 2:** Changes in liver stiffness according to breathing phases.

	**Intercostal**	**Subcostal**	***p*** **-value**
FE 10 mL/kg	0.33 ± 0.34 kPa	0.37 ± 0.35 kPa	0.801
FE 5 mL/kg	0.29 ± 0.31 kPa	0.21 ± 0.17 kPa	0.530
FI 5 cm H_2_O	0.17 ± 0.10 kPa	0.35 ± 0.23 kPa	0.044
FI 10 cm H_2_O	0.50 ± 0.45 kPa	1.16 ± 0.49 kPa	0.022
FI 15 cm H_2_O	1.03 ± 0.64 kPa	2.18 ± 0.96 kPa	0.028
FI 20 cm H_2_O	1.90 ± 0.84 kPa	3.03 ± 1.07 kPa	0.017

*All data are presented as mean ± standard deviation*.

*p < 0.05 is considered statistically significant*.

*FE, forced-expiration; FI, forced-inspiration*.

## Discussion

SWE is a robust technique that allows the measurement of liver stiffness and the assessment of hepatic fibrosis in a non-invasive manner (25). In human medicine, the effects of various confounding factors, such as the probe used, patient positioning, fasting, ROI, hepatic lobe, anesthesia, breathing, and scanning approach on the measurement of liver stiffness, have been examined (7). In elastographic examinations of the liver in humans, a convex probe is typically applied in adults, while a linear probe can be used in pediatric patients owing to the limited amount of superficial fat tissue (26). In contrast to the situation in humans, the canine liver is easily visualized with a linear probe, and convex probes are typically not used. As such, the difference in liver stiffness measurements between linear and convex probes was not evaluated in this study. In humans, liver stiffness differs significantly between relaxed and stretched arm positions (7). However, weak restraints in dogs similar to the relaxed arm position permit movement and are thus difficult to apply in practice. Furthermore, liver stiffness was not compared between post-prandial and fasted states in this study because fasting is usually recommended prior to US examination in dogs (27). Studies in the veterinary literature have reported the effects of ROI, hepatic lobe, and scanning approach on liver stiffness (10). However, the effects of the interaction between scanning approach and breathing on liver stiffness have not been investigated. Further, confounding factors such as anesthesia and breathing have not been evaluated in dogs. Therefore, this study investigated the effects of anesthesia, breathing, and scanning approach on liver stiffness when performing 2D-SWE in dogs. The results of this study confirm no significant difference in liver stiffness between conscious and anesthetized states, and between free-breathing and breath-holding conditions. Liver stiffness was significantly different between breathing phases, especially in forced-inspiratory phases with high airway pressure, and between scanning approaches.

In young children, transient elastography should be performed in combination with sedation to avoid unintended movements that lead to invalid measurements (11). In this regard, previous studies in dogs have suggested that sedation or anesthesia may be necessary to reduce motion during data acquisition (10, 28). In a previous human study, liver stiffness in children was significantly higher during anesthesia than in the awake state (9). Conversely, no significant differences were observed in liver stiffness between conscious and anesthetized states in this study. The discrepant results between humans and dogs may be attributed to the different types of anesthetic agents used. While the anesthetic drugs used in humans have not been comprehensively reported in the literature, propofol induction and isoflurane maintenance were performed in the present study. In dogs, propofol increases total hepatic blood flow (29), whereas isoflurane decreases it (30). Given that an increase in hepatic blood flow leads to higher liver stiffness (20), the current results could be due to the counteractive effects of propofol and isoflurane on liver stiffness. Although further research is required to identify the impact of various anesthetic drugs on liver stiffness, hepatic 2D-SWE can be performed in uncooperative dogs under general anesthesia with propofol and isoflurane, as the stiffness is less strongly influenced by the combined use of these drugs.

In young children who cannot hold their breath properly, hepatic SWE is often performed during free-breathing. Conflicting results have been reported with regard to differences in liver stiffness between free-breathing and breath-holding conditions. Some studies have reported no significant difference (5, 12, 14, 16, 17), whereas others have indicated that liver stiffness is lower in free-breathing conditions than in breath-holding conditions (13, 15). Since expiratory time is twice as long as inspiratory time (31), several veterinary studies have attempted to perform hepatic 2D-SWE at end-expiration to minimize the effects of breathing motion artifacts (8, 10, 32). However, in clinical settings, irregular breathing in dogs makes it challenging to perform 2D-SWE at the exact end-expiration. As breathing motion artifacts did not significantly affect liver stiffness in this study, no additional adjustments were required to mitigate the effects of breathing motion artifacts during free-breathing in dogs. In human studies, the time to acquire data during hepatic SWE was longer in breath-holding conditions than in free-breathing conditions (14). Similarly, additional time is required for breath-holding conditions in dogs, since this condition is induced by blowing air into, or holding, the nose and mouth. Therefore, given that no significant difference was observed in liver stiffness between the two conditions, free-breathing examination in dogs with normal breathing cycles may be advantageous in terms of time expenditure. However, excessively rapid respiratory rates can lead to non-stabilized and less saturated 2D-SWE boxes, resulting in inaccurate estimation of liver stiffness (13, 15). Rapid respiratory rates are observed in panting dogs that experience respiratory problems or are excited during the examinations; in such situations, the breath-holding technique may be more suitable.

Liver stiffness was significantly higher in forced-inspiration with high airway pressure than in other breathing phases. Similar to the current findings, liver stiffness measured using magnetic resonance elastography was higher in inspiratory breath-holding than in expiratory breath-holding states (19, 20), and deep inspiration significantly increased liver stiffness in US elastography (3, 18). Two potential explanations for the increase in liver stiffness during deep inspiration include mechanical compression (19) and hemodynamic compression (20). With regard to mechanical compression, the diaphragm compresses the liver parenchyma and causes hepatic deformation during inspiration, leading to increased liver stiffness. In contrast, in hemodynamic compression, the diaphragm compresses the hepatic vein and reduces blood return during inspiration, thereby increasing hepatic venous pressure, which leads to elevated liver stiffness. Therefore, since deep inspiration significantly affects liver stiffness, this breathing phase should be avoided when performing hepatic 2D-SWE in dogs.

In this study, no significant difference was noted in liver stiffness among all expiratory phases and inspiratory phases with low airway pressure. This is in contrast to previous human studies that reported higher liver stiffness in expiration than in normal gentle inspiration (21, 22). However, these studies utilized transient elastography and point shear wave elastography, which have lower reliability and accuracy for measuring liver stiffness compared with 2D-SWE that was used in the present study (33–37). In contrast to transient elastography that lacks a B-mode image and point shear wave elastography that does not provide an image of stiffness, 2D-SWE provides both these images by presenting anatomical and tissue stiffness information through visualization of a color box on a B-mode image (6). Accordingly, the results of the present study are considered to be more reliable than the previous human studies. A possible reason underpinning the lack of a significant difference is that the degree of inspiration and expiration may have been insufficient to induce mechanical and hemodynamic changes to increase liver stiffness. In the same context, a previous study reported that the changes in liver volume were <3% during a normal breathing cycle (38). This suggests that hepatic 2D-SWE can be performed at any breathing phase in dogs with stable breathing.

Liver stiffness measurements were significantly higher in the subcostal approach than in the intercostal approach. This is contrary to the results of a previous study on Beagle dogs, which reported no significant difference in liver stiffness between intercostal and subcostal approaches (10). In humans, the subcostal approach is known to demonstrate a false elevation in liver stiffness due to probe compression artifacts (39). Thus, the intercostal approach is recommended because it can be performed with less compression (40). Since Beagles are deep-chested breeds, the subcostal approach may have induced probe compression artifacts, which could have resulted in increased liver stiffness. Therefore, the intercostal approach may be more appropriate when measuring liver stiffness in dogs, similar to that in humans. This is especially true when assessing the right lobe of the liver in deep-chested dogs, whereby the right intercostal approach is more advantageous than the subcostal approach (41).

There was a significant effect of the interaction between scanning approach and breathing phase on liver stiffness. In all inspiratory phases, changes in liver stiffness were significantly higher in the subcostal approach than in the intercostal approach. This suggests that the effect on breathing is greater for the subcostal approach than the intercostal approach. As mentioned above, probe compression artifacts in the subcostal approach may have underpinned the increase in liver stiffness, but this does not fully explain the greater changes in liver stiffness in terms of breathing phases in the subcostal approach. A human study indicated that the liver moved more in the superior-inferior direction than in the left-right direction (42), suggesting that the diaphragm may have compressed the liver in the superior-inferior direction to induce severe deformity in parts of the liver lobes that were compressed. In this regard, it is plausible that the difference in changes in liver stiffness was due to mechanical compression, because the liver lobes visualized using the subcostal approach appeared more deformed than those visualized using the intercostal approach. Therefore, examination using the intercostal approach is recommended for hepatic 2D-SWE in dogs, given the smaller influence of breathing phase on liver stiffness compared with that in the subcostal approach.

This study had several limitations. First, it was performed with a limited number of dogs, and only male Beagle dogs with similar body weight and age were included. Further studies with a larger number of subjects, various breeds, sexes, body weights, and ages are warranted. Second, cytological and histopathological examinations to exclude the possibility of liver disease were not performed. However, the Beagle dogs in this study were relatively young and exhibited no clinical signs of illness and abnormalities on physical examination, blood examination, radiography, and ultrasonography. Third, dogs with hepatic fibrosis were not included. In human elastography, liver stiffness differs significantly between the inspiratory and expiratory phases in patients with liver fibrosis, but not in healthy individuals (20). Fourth, the forced-expiratory volume applied in this study was set at 5 and 10 mL/kg. However, it remains unclear whether these volumes truly represented the normal physiological expiratory pressure. Further studies are required to analyze the relationship between the artificial forced-expiration and the normal physiologic expiration. Finally, intra- and inter- observer reliability were not evaluated, which should be further assessed while considering various confounding factors.

In conclusion, this study demonstrates that 2D-SWE is capable of measuring liver stiffness in dogs without being confounded by the effects of anesthesia and free-breathing. Further, deep inspiratory phases including 15 and 20 cm H_2_O in the intercostal approach and 10, 15, and 20 cm H_2_O in the subcostal approach falsely increase liver stiffness. In terms of scanning approach, the intercostal approach may reduce undesired increases in liver stiffness. Therefore, when performing 2D-SWE in dogs, liver stiffness should be interpreted considering these confounding factors.

## Data Availability Statement

The raw data supporting the conclusions of this article will be made available by the authors, without undue reservation.

## Ethics Statement

The animal study was reviewed and approved by Institutional Animal Care and Use Committee of Konkuk University (approval no. KU 21127).

## Author Contributions

JC contributed to the study design, data collection, data interpretation, and manuscript writing. JayK and JaeeK contributed to the data collection and data interpretation. JaehK and KE contributed to the study design, data interpretation, and manuscript editing. All authors reviewed and approved the final submitted manuscript.

## Conflict of Interest

The authors declare that the research was conducted in the absence of any commercial or financial relationships that could be construed as a potential conflict of interest.

## Publisher's Note

All claims expressed in this article are solely those of the authors and do not necessarily represent those of their affiliated organizations, or those of the publisher, the editors and the reviewers. Any product that may be evaluated in this article, or claim that may be made by its manufacturer, is not guaranteed or endorsed by the publisher.

## Supplementary Material

Supplementary Video 1 A video of hepatic 2D-SWE in a Beagle dog during forced-expiration.

Supplementary Video 2 A video of hepatic 2D-SWE in a Beagle dog during end-expiration.

Supplementary Video 3 A video of hepatic 2D-SWE in a Beagle dog during forced-inspiration.
